# Prevalence of Valvular and Non-valvular Atrial Fibrillation and the Application of Antithrombotic Treatment in a Tertiary Care Hospital

**DOI:** 10.31729/jnma.5113

**Published:** 2020-11-30

**Authors:** Sahadeb Prasad Dhungana, Rinku Ghimire

**Affiliations:** 1 Cardiology Unit, Department of Internal Medicine, Nobel Medical College Teaching Hospital, Biratagar, Nepal; 2 Department of Pharmacology, Nobel Medical College Teaching Hospital, Biratagar, Nepal

**Keywords:** *anticoagulants*, *atrial fibrillation*, *risk assessment*, *stroke*

## Abstract

**Introduction:**

Atrial fibrillation is a common atrial tachyarrhythmia with an increased risk of throm-boembolism. This study aims to provide information about the application of antithrombotic treatment based on risk stratification schemes for stroke in real-life clinical practices.

**Methods:**

This was a descriptive cross-sectional study in 260 patients admitted at the tertiary care hospital with a diagnosis of atrial fibrillation from January 2019 to February 2020 after approval from the Institutional Review Committee (ref. no. 207/2018). Convenient sampling was used. Predisposing conditions for atrial fibrillation, risk factors for stroke, and the use of antithrombotics were obtained based on the pre-structured questionnaires. Data were analyzed by Statistical Package for the Social Sciences version 20.

**Results:**

The prevalence of valvular and non-valvular atrial fibrillation was 125 (48.0%), and 135 (51.9%) respectively. Among patients with a non-valvular variant, 102 (75.5%) had a CHA_2_DS_2_VASc-score of ≥2 who were eligible for oral anticoagulants, 13 (9.6%) patients received it with a majority having sub-therapeutic international normalized ratio. Among patients with valvular type, only 47 (37.6%) patients were receiving oral anticoagulants and 20 (42.5%) patients achieved therapeutic international normalized ratio. Two hundred forty three (93.4%) patients had dilated left atrium (≥40mm), 119 (45.9%) had hypertension and 27 (10.3%) had diabetes mellitus.

**Conclusions:**

Antithrombotics were markedly underused in patients with atrial fibrillation. There is a need for proper application of risk stratification schemes for stroke and appropriate use of anti-thrombotics to prevent thromboembolism.

## INTRODUCTION

Atrial fibrillation (AF) is the most common sustained tachyarrhythmia which affects 1-2% of the general population.^1^ AF is a risk factor for stroke and thromboembolism. There is higher mortality and morbidity when a stroke occurs in association with AF. Therefore, stroke prevention is a major concern when managing patients with AF. Various clinical risk factors have been identified, which confer a high risk of stroke.

An important aspect of AF management is the prevention of stroke, using antithrombotic agents including anti-platelets and oral anticoagulants (OACs).^2^ Although the literature provides simplified rules for the use of antithrombotic in patients with AF, studies have shown that OAC is frequently underused in AF patients with reported use in between 30 and 60%.^1,3,4^

This suboptimal use may be related to various physician and patient factors. This study provides information about the application of antithrombotic treatment for patients with valvular atrial fibrillation (VAF) and non-valvular atrial fibrillation (NAVF) in a tertiary care hospital.

## METHODS

This descriptive cross-sectional study was conducted from January 2019 to February 2020. A total of 260 patients with AF (age ≥15 years) who attended the cardiology clinic or admitted to the cardiology unit of Nobel Medical College Teaching Hospital were enrolled based on a convenient sampling method. Ethical approval was obtained from the institutional review committee (NMCTH ref. no. 207/2018) before starting the study. The sample size (n) was calculated using the formula,

$$\begin{array}{ll} \text{n} & \text{= }{\text{Z}}^{\text{2}}\times \text{p}\times \text{(1}-\text{p)}/{\text{e}}^{\text{2}}\\ & \text{= }{\left(1.96\right)}^{\text{2}}\times 0.138\times 0.86/{(\text{0.05)}}^{\text{2}}\\ & \text{= }185 \end{array}$$

Where,

n = sample size
Z = 1.96 at 95% Confidence Interval (CI)
p = prevalence of AF, 13.8%^6^
e = margin of error, 5%

Taking a 10% non-respondent rate, the sample size becomes 204. However, the total sample size was 260.

All participants were asked relevant questions to note demographic information, co-morbid conditions, use of antithrombotic agents, and risk factors for stroke based on pre-structured questionnaires. The international normalized ratio (INR) value of patients who were on vitamin K antagonists (VKA) was noted at the time of enrollment. CHA_2_DS_2_-VASc (Congestive heart failure, Hypertension, Age ≥75 years, Diabetes mellitus, prior stroke or transient ischemic attack, vascular disease, age 65-74, female sex) score was determined by using age and co-morbidities at the time of enrollment for stratifying stroke risk.^5^ Electrocardiogram (ECG) and echocardiogram (Echo) were performed in each case.

Data were entered in Microsoft excel 2007 and converted into IBM Statistical Package for the Social Sciences (SPSS), version 20. Continuous and categorical variables were presented as mean, percentage, and range. The tabular presentation was made for necessary variables.

## RESULTS

The prevalence of valvular AF is 125 (48.0%) and non-valvular AF is 135 (51.9%). The majority of patients 156 (60%) were of age >60 years. The mean heart rate was 95.65 beats per minute. Left atrial/ left atrial appendage clot was seen in 30 (11.53%) patients (Table 1).

**Table 1:** Baseline characteristics of patients with atrial fibrillation (n = 260).

Characteristics	Frequency n (%)
Non-valvular AF	135 (51.9)
Valvular AF	125 (48.0)
Rheumatic heart disease: Mitral Stenosis	120 (46.1)
Mechanical mitral valve replacement	5 (1.9)
Male	114 (43.8)
Female	146 (56.1)
Smoker	89 (34.2)
Alcohol consumer	32 (12.3)
Heart rate (BPM)	
<100	151 (58.0)
>100	109 (41.9)
LA/LAA clot	30 (11.53)
Mean values	
Mean BMI in kg/m^2^ (Range)	23.62 (13.02-39)
Mean systolic blood pressure in mmHg (Range)	119.60 (80-180)
Mean diastolic blood pressure in mmHg (Range)	77.06 (50-110)
Mean hemoglobin (gm/dl) (Range)	12.38 (8.3-18.7)
Mean serum creatinine (mg/dl)	0.93 (0.6-1.6)
Mean heart rate (BPM)	95.65 (50-160)
Mean CHA_2_DS_2_-VASc score in NVAF (Range)	1.9 (0-5)

Abbreviations: BMI; body mass index, BPM; beat per minute, AF; atrial fibrillation, LA; left atrium, LAA; left atrial appendage, NVAF; non-valvular atrial fibrillation, CHA_2_DS_2_-VASc-Congestive heart failure, Hypertension, Age ≥75 years, Diabetes mellitus, prior Stroke or transient ischemic attack, vascular disease, age 65-74, female sex

Among predisposing conditions for NVAF: left ventricular (LV) diastolic dysfunction in 87 (33.4%), LV systolic dysfunction 84 (32.3%), overweight or obesity 73 (28.0%), coronary artery disease (CAD) 27 (10.3%), hypertension with left ventricular hypertrophy (LVH) 25 (9.6%), dilated cardiomyopathy (DCM) 17 (6.5%), chronic obstructive airway disease (COPD) and/or corpulmonale in 37 (14.2%) were common (Table 2).

**Table 2:** Predisposing conditions for atrial fibrillation (n = 260).

Characteristics	Frequency n (%)
Dilated LA	231 (88.8)
RHD	125 (48)
Smoking	89 (34.2)
LV diastolic dysfunction	87 (33.4)
LV systolic dysfunction	84 (32.3)
Overweight/obesity	73 (28.0)
Alcohol use	32 (12.3)
CAD	27 (10.3)
LVH	25 (9.6)
COPD	19 (7.3)
Cor pulmonale	18 (6.9)
DCM	17 (6.5)
Lone AF	13 (5)
Thyroid disorders	3 (1.1)

Abbreviations: LA; left atrium, RHD; rheumatic heart disease, LV; left ventricle, CAD; coronary artery disease, LVH; left ventricular hypertrophy, COPD; chronic obstructive pulmonary disease, DCM; dilated cardiomyopathy, AF; atrial fibrillation

Risk factors for stroke in patients with NVAF are illustrated below (Table 3).

**Table 3:** Risk factors for stroke in non-valvular atrial fibrillation (n = 135).

Risk factors for stroke	Frequency n (%)
Age	
65 -74 years	53 (39.2)
>75 years	42 (31.1)
Prior stroke or TIA	20 (14.8)
CHF or LVEF <40%	64 (47.4)
Hypertension	62 (45.9)
Diabetes Mellitus	14 (10.3)
CHA_2_DS_2_-VASc score	
0	9 (6.6)
1	24 (17.7)
2	41 (30.3)
3	52 (38.5)
4	7 (5.1)
5	2 (1.48)
LA size (mm)	
>40mm	120 (88.8)
≤40mm	15 (11.1)

Abbreviation: TIA; transient ischemic attack, CHF; chronic heart failure, LVEF; left ventricular ejection fraction, LA ; left atrium,CHA_2_DS_2_-VASc - Congestive heart failure, Hypertension, Age ≥75 years, Diabetes mellitus, prior Stroke or transient ischemic attack, vascular disease, age 65-74, female sex

Among echocardiogram parameters, 88.8% patients had dilated LA, 135 (51.9%) of patients had NVAF, 87 (33.4%) had diastolic dysfunction, 84 (32.3%) had systolic dysfunction, and 33% pulmonary artery hypertension. AF with fast ventricular rate 109 (41.9%), LVH 25 (15.8%), right ventricular hypertrophy 26 (10%), left bundle branch block 16 (8.3%) and right bundle branch block 8 (3.3%) were the common ECG findings.

Anticoagulant prophylaxis according to each CHA_2_DS_2_-VASc score is summarized below (Table 4).

**Table 4:** Antithrombotic treatment in Non-valvular AF according to CHA_2_DS_2_-VASc score (n = 135).

CHA_2_DS_2_-VASc score	Frequency n (%)	Aspirin	Warfarin	None
0	9 (6.6)	8	0	1
1	24 (17.7)	18	0	6
2	41 (30.3)	26	2	13
3	52 (38.5)	31	7	14
4	7 (5.1)	4	3	0
5	2 (1.4)	0	1	1

Abbreviations: CHA_2_DS_2_-VASc-Congestive heart failure, Hypertension, Age ≥75 years, Diabetes mellitus, prior Stroke or transient ischemic attack, vascular disease, age 65-74, female sex

Among 135 patients with NVAF, 102(75.5%) had a CHA_2_DS_2_-VASc score of ≥2 who were eligible for oral anticoagulants, 13 (9.6%) patients received it with a majority having sub-therapeutic INR. Among 125 (48.0%) patients with VAF, only 47 (37.6%) patients were receiving oral anticoagulants and 20 (42.5%) patients achieved therapeutic INR (Table 5). The majority of patients with VAF or NVAF were receiving Aspirin as an antithrombotic agent.

**Table 5:** The number of patients with therapeutic INR (between 2 and 3).

Characteristics	No. of patients on VKA n (%)	No. of patients with therapeutic INR n (%)
Total	60 (23.07)	25 (41.6)
Valvular AF	47 (37.6)	20 (42.5)
Non-valvular AF	13 (9.6)	5 (38.4)

Abbreviations: AF; atrial fibrillation, VKA; vitamin K antagonist, INR; International normalized ratio

## DISCUSSION

AF is the most common sustained cardiac tachyarrhythmia and a major risk factor for thromboembolism.^7^ Antithrombotic agents is highly effective for the reduction of thromboembolism and stroke in patients with AF. VKA is more effective than aspirin with a relative risk reduction of 36%.^8^

This study has shown the underuse of antithrombotic therapy in the form of either OACs or antiplatelet agents in patients with both VAF and NVAF according to contemporary international guidelines.^9^ About 37.6% of patients with VAF and 9.6% of patients with NVAF received OACs. The majority of patients with both VAF and NVAF were receiving Aspirin as an antithrombotic agent. Likewise, a study done in a rural part of Nepal by the same author showed that 39.1% of patients with NVAF who had a CHA_2_DS_2_ score of > 2 and eligible for oral anticoagulants, only 18.9% of patients received it. Similarly, only 22.7% of patients with valvular AF obtained OACs.^10^

The stroke risk in patients with NVAF with a high score corresponds to greater risk while a low score corresponds to a lower risk.^6^ The European Society of Cardiology (ESC)^11^ and National Institute for Health and Care Excellence (NICE)^12^ guidelines recommend that if the patient has a CHA_2_DS_2_-VASc score of two and above, OAC is recommended. In our study, among 135 patients with non-valvular AF, 75.5% had a CHA_2_DS_2_-VASc score of ≥ 2 who were eligible for oral anticoagulants, only 9.6% of patients received it with the majority having sub-therapeutic INR.

Most of the discussions about the risk of stroke are related to NVAF from the western world. Owing to the high burden of RHD in our part of the world, we have a good number of mitral stenosis (MS) which increases the risk of stroke even in absence of AF, and the risk is increased markedly in the presence of AF. The risk of stroke is increased 17 fold in patients with AF and RHD.^13^ In our study, 48.0% of total patients with AF had RHD with MS and 37.6% of these patients were on OACs. Only 42.5% of patients who were receiving OACs attained therapeutic INR. This indicates the inadequate use of this highly effective anticoagulant therapy among patients with VAF who are at high risk for stroke.

Some of the patient factors related to OAC underuse could be a lack of awareness or need for monthly INR monitoring with the use of VKA. The reasons for under-treatment seem complex but may include lack of knowledge about trials or guidelines among treating physicians, bleeding risk, poor drug compliance, cost of treatment, inconvenience of INR monitoring, unavailability and high cost of newer direct oral anticoagulants (DOACs). Continuing efforts should be made to improve patients' awareness and understanding of the disease process as well as the need for OAC therapy.^14^

Education to health personnel and patients on stroke prevention from AF may improve the prescription rate of antithrombotic therapy. The stroke risk stratification scheme may help to determine which drug is most appropriate for different risk category patients. To facilitate the tailored treatment, guideline formulation group and physicians should focus on providing uniform and easy to use risk stratification tool. Patient's knowledge about antithrombotic therapy for AF might be improved by an information booklet.^15^ There is still an unmet need for safer and easier to use antithrombotic drugs in Nepal.

This is a small cross-sectional study representing patients from the eastern part of Nepal. The therapeutic INR values may not be appropriate for comparison with other studies because a single INR value was noted at the time of enrollment. We found the marked underuse of antithrombotic in patients with AF but we did not look at the physician's and patient's factors for not prescribing antithrombotic treatment. Bleeding risk scores were not taken into account. Since DOACs are not easily available or costly in our country so data on the use of DOACs is not presented in this study.

## CONCLUSIONS

This study provides information about current practices of the use of antithrombotic agents and stroke risk in our patient's population with AF. OACs were markedly underused who qualified for it. This indicates the need for appropriate use of OACs to prevent thromboembolism and stroke. Improved awareness and understanding of the perceived risks of underutilization of antithrombotics among physicians and patients may improve its use. Specialized anticoagulation clinics and laboratory services at the grass-root health care center may help to initiate and maintain safe oral anticoagulant therapy.
